# Are participants with disability referred for social prescribing? Findings from the English Longitudinal Study of Ageing

**DOI:** 10.1093/pubmed/fdaf143

**Published:** 2025-11-03

**Authors:** Laurie E Davies, David R Sinclair, Angel Osei, Andrew Kingston, Mark Adley, Orla Whitehead, Barbara Hanratty

**Affiliations:** Population Health Sciences Institute, Newcastle University, Campus for Ageing and Vitality, Biomedical Research Building (Second floor), Westgate Road, Newcastle upon Tyne, NE4 5PL, UK; Population Health Sciences Institute, Newcastle University, Campus for Ageing and Vitality, Biomedical Research Building (Second floor), Westgate Road, Newcastle upon Tyne, NE4 5PL, UK; School of Pharmacy, Newcastle University, King George VI Building, King's Rd, Newcastle upon Tyne, NE1 7RU, UK; Population Health Sciences Institute, Newcastle University, Campus for Ageing and Vitality, Biomedical Research Building (Second floor), Westgate Road, Newcastle upon Tyne, NE4 5PL, UK; Population Health Sciences Institute, Newcastle University, Campus for Ageing and Vitality, Biomedical Research Building (Second floor), Westgate Road, Newcastle upon Tyne, NE4 5PL, UK; Population Health Sciences Institute, Newcastle University, Campus for Ageing and Vitality, Biomedical Research Building (Second floor), Westgate Road, Newcastle upon Tyne, NE4 5PL, UK; Population Health Sciences Institute, Newcastle University, Campus for Ageing and Vitality, Biomedical Research Building (Second floor), Westgate Road, Newcastle upon Tyne, NE4 5PL, UK

**Keywords:** disabilities, primary care, ageing

## Abstract

**Background:**

Social prescribing connects NHS patients to activities, groups, and services in their community to address wider health determinants. Those living with disability may have needs for support from social prescribing, yet there is little evidence exploring this.

**Methods:**

We examined the association between referral to social prescribing and disability, using logistic regression in 5578 individuals aged ≥50 from wave 10 of the English Longitudinal Study of Ageing (year 2021–23).

**Results:**

Referral to social prescribing is significantly associated with mobility disability (OR 1.68 [95% CI: 1.26–2.23]), Instrumental Activities of Daily Living disability (1.56 [1.16–2.09]) and basic Activities of Daily Living disability (1.39 [1.03–1.86]). Our results suggest that this is driven by referrals for: (i) participants at the earlier stages of disability (i.e. with difficulty climbing stairs (1.49 [1.13–1.97]), walking ¼ mile unaided (1.83 [1.37–2.44]), and doing [heavy] housework (1.47 [1.08–1.98]), (ii) participants with difficulty managing money (1.85 [1.06–3.08]), and (iii) those with difficulty dressing (1.47 [1.08–1.99]) or using the toilet (1.72 [1.12–2.59]).

**Conclusions:**

Older adults at either end of the disability spectrum are referred to social prescribing. Further research is needed to understand whether this is meeting needs, and if benefits vary with the level or nature of the disability.

## Background

Social prescribing describes an holistic approach for linking National Health Service (NHS) patients to activities, groups and services in their community, to help them (i) address their wider determinants of health (such as issues with housing, food, employment, income or social support), (ii) modify their health behaviours, and (iii) better manage their conditions.^1^^,^^2^ The interventions offered are wide-ranging, and cover the arts (e.g. dance, crafts), exercise (e.g. classes, clubs), the outdoors (e.g. gardening, walking groups), welfare advice (e.g. for debt, housing, benefits or unemployment), and other forms of assistance (e.g. befriending, and healthy eating advice).^3–6^ Social prescribing is not a new idea, but has gained more traction over recent years. For example, as of 2024 in England, over 2.5 million referrals have been made to social prescribing link workers (with >3500 of them in post).^7^ This reflects an NHS commitment to universal personalized care and access to social prescribing for all, via general practice.^3^

The impact of social factors on health is well recognized.^8^ Previous studies of social prescribing have included people with conditions such as type 2 diabetes,^9^ loneliness^10^ and depression,^11^ but the evidence base is sparse overall.^12^ In particular, there is a dearth of literature concerning whether social prescribing is associated with disability, which usually begins with mobility difficulty (e.g. using steps, walking 400 yards), followed by impairment in household care (e.g. meal preparation, housework), then basic personal care (e.g. dressing, eating).^13–15^ This evidence gap is despite the large number of people living with disability in England,^16^ and the research to suggest that changes in finances following the acquisition of disability have a detrimental effect on housing, transport, social interactions, and personal relationships^17^ (which might potentially be helped through social prescribing).

Therefore, this study aims to determine whether adults in England living with disability are being referred to social prescribing services by analysing primary care referral patterns recorded in wave 10 of the English Longitudinal Study of Ageing (ELSA).

## Methods

### Participants

We used data from the most recent wave of ELSA (wave 10, 2021–23) at the time of conducting this research.^18^ Beginning in 2002, ELSA is an ongoing biennial study, drawn from a nationally representative sample (Health Survey for England), that remains representative of adults aged ≥50 in England due to periodic sample refreshment.^19^^,^^20^ From an initial sample of 7482 participants aged ≥50, 5578 participants (4742 core members, 836 partners) with complete data for social prescribing, disability and confounding variables, formed the basis of this analysis. We included both core members (who constitute the original sample and are weighted to be representative of the nation) and their cohabiting partners (who join after initial recruitment to provide a fuller household context but are not included in the survey weighting) in view of the limited number of referrals for social prescribing.

### Variable definitions

We defined social prescribing by an affirmative response to whether a doctor, social worker or other health professional had referred participants to take part in any of the following: (i) arts, crafts, music, reading groups, or social groups, (ii) a gardening or nature activity, (iii) outdoor health or fitness activities/clubs, (iv) indoor exercise or other clubs/activities, (v) adult learning or skills development training, (vi) employment or benefit support, and/or (vii) another social, community or volunteering activity.

According to the binary format of the ELSA disability questions,^21^ we defined mobility disability, Instrumental Activities of Daily Living (IADL) disability, and basic Activities of Daily Living (ADL) disability based on participants’ self-reporting ‘any difficulty’ (yes/no) with at least one of the activities in Table 1. We closely matched these activities to those previously reported^13^ in order to examine whether referrals for social prescribing reflect the hierarchical loss of disability,^13^ with some exceptions: (i) We used difficulty walking across a room (which captures individuals with more severe mobility impairment, who are likely to require immediate support or assistive technology)^22^ in place of ‘difficulty moving around the house’ (which was not available).^13^ (ii) As ‘difficulty doing work around the house/garden’ does not disaggregate heavy from light housework, we used difficulty pushing/pulling large objects (as a proxy for heavy housework, i.e. vacuuming) and difficulty reaching or extending arms above shoulder level (as a proxy for light housework, i.e. dusting) in a sensitivity analysis. (iii) In contrast to a previous study,^13^ we included difficulty managing money and taking medications in our disability definition, as they are relevant to the context of examining social prescribing. For example, difficulty managing money—while not necessarily clinically related to executive dysfunction—can be a common reason for referral to services such as employment and benefits support. (iv) Within our ADL definition, we did not include the ELSA survey item ‘difficulty getting up from a chair after sitting for long periods’ as a measure of chair transfer ability, because the ‘after sitting for long periods’ qualifier could pick up more general stiffness or mild limitations rather than profound disability. (v) ‘Difficulty cutting toenails’ is the first ability to be lost in the disablement process,^13^^,^^14^ but was not available in ELSA. Hence, we examined ‘difficulty stooping, kneeling, or crouching’ as a potential proxy measure. We did so as these items require similar abilities (balance in unstable postures) to those needed for toenail cutting.^13–15^ However, we did not include ‘difficulty stooping, kneeling, or crouching’ within our composite mobility, IADL, and ADL definitions. The reason for this exclusion was because we could not validate it as a proxy measure, given that data for difficulty cutting toenails was not collected.

**Table 1:** Disability definitions

Mobility disability	Difficulty walking across a room, walking ¼ mile (0.4 km) unaided or climbing stairs.
IADL disability	Difficulty preparing a hot meal, shopping, doing work around the house/garden, managing money or taking medication
ADL disability	Difficulty getting or/out of bed, using the toilet, dressing, bathing or eating.

The confounding variables outlined in Appendix Table 1 included age, sex, disease count, loneliness, and total net non-pension wealth quintiles—based on the whole sample—as a robust indicator of socioeconomic position, reflecting past and present circumstances.^23^^,^^24^

### Statistical analysis

We used unweighted logistic regression in R version 3.6.0 to examine whether social prescribing was associated with disability, accounting for confounding variables. We took this approach in line with the ELSA user guide, which says that *‘If non-core sample members are to be analysed they should be analysed unweighted’.* Therefore, findings should be interpreted as within-sample associations rather than population-representative estimates.

## Results

### Participant characteristics

The complete case sample comprised 5578 participants (3117 women and 2461 men), of mean age 68.1 years (SD 9.4). Only 5.7% of participants (319/5578) were referred for social prescribing. Most participants had three conditions (IQR2–4), and a quarter (25.2%) of participants were lonely (*n* = 1404/5578). Over a third (35.1%) of participants had mobility disability (*n* = 1960/5578), 14.8% (n = 828/5578) had IADL disability, and 15.8% (*n* = 882/5578) had ADL disability. Of the component disability items, participants often had difficulty climbing stairs (29.5%, 1645/5578), walking a quarter of a mile unaided (25.1%, 1399/5578), doing work around the house/garden (12.9%, 719/5578) and dressing (12.1% (673/5578), but infrequently reported difficulty with other activities (Appendix Table 2).

### Logistic regression

Social prescribing is significantly associated with mobility, IADL, and ADL disability. Our results suggest that this is driven by three things: (i) referrals for participants at the earlier stages of disability (i.e. with difficulty climbing stairs, walking ¼ mile unaided, and doing [heavy] housework), (ii) referrals for participants with difficulty managing money, and (iii) by referrals for difficulty dressing or toileting. The effect size for housework is indicative of heavy housework, as in our sensitivity analysis, participants with difficulty pulling or pushing large objects (which could be indicative of vacuuming) were referred for social prescribing, whereas those with difficulty reaching or extending arms above the shoulder level, which is indicative of light housework activities like dusting, were not. Participants with difficulty stooping/kneeling/crouching (which suggests early-stage disability)^15^ were also referred for social prescribing, as were those in the poorest wealth quintile, and those with loneliness or long-term conditions (Table 2).

**Table 2:** Associations between social prescribing and disability independent of confounding factors

		Odds ratio (95% confidence interval) for association with the binary social prescribing variable^e^
Binary disability items^a^	Mobility	**1.68 (1.26–2.23)**
	IADL	**1.56 (1.16–2.09)**
	ADL	**1.39 (1.03–1.86)**
Individual ADL items^a^	Dressing	**1.47 (1.08–1.99)**
	Bathing	1.27 (0.87–1.83)
	Eating	1.27 (0.66–2.26)
	Getting in/out bed	0.91 (0.59–1.38)
	Going to the toilet	**1.72 (1.12–2.59)**
Individual IADL items^a^	Preparing a meal	1.47 (0.93–2.23)
	Shopping	1.20 (0.81–1.73)
	Taking medication	1.54 (0.76–2.86)
	Doing work around the house/garden	**1.47 (1.08–1.98)**
	Managing money	**1.85 (1.06–3.08)**
Individual mobility items^a^	Walking across a room	1.12 (0.67–1.79)
	Climbing stairs	**1.49 (1.13–1.97)**
	Walking a quarter of a mile unaided	**1.83 (1.37–2.44)**
Sensitivity analysis for housework^a^	Pushing/pulling large objects (heavy housework proxy i.e. vacuuming)	**1.40 (1.04–1.89)**
	Reaching or extending arms above shoulder level (light housework proxy i.e. dusting)	1.10 (0.77–1.54)
Proxy for difficulty cutting toenails^a^	Difficulty stooping/kneeling/crouching (requiring balance in unstable postures)	**1.36 (1.05–1.77)**
Wealth quintiles (referenced to quintile 3)^b^	Quintile 1 (poorest)	**1.57 (1.10–2.27)**
	Quintile 2	0.91 (0.61–1.36)
	Quintile 4	1.14 (0.77–1.68)
	Quintile 5 (wealthiest)	1.28 (0.88–1.89)
Loneliness^c^	Loneliness	**1.81 (1.42–2.30)**
Disease count^d^	Each additional disease	**1.11 (1.04–1.19)**

a Adjusted for age, sex, total net non-pension wealth quintiles, loneliness and disease count

b Adjusted for age, sex, binary disability, loneliness and disease count

c Adjusted for age, sex, binary disability, total net non-pension wealth quintiles and disease count

d Adjusted for age, sex, binary disability, total net non-pension wealth quintiles and loneliness

e Bold text represents statistically significant results.

## Discussion

### Main finding of this study

Participants at either end of the disability spectrum, those with difficulty managing money, and the poorest are most likely to be referred for social prescribing.

### What is already known on this topic, and what this study adds

In this study, participants with difficulty performing activities that occur towards the start of the disablement process, such as using steps, walking 400 yards and stooping, kneeling or crouching,^13^ were referred for social prescribing. As all of these activities require long-distance mobility and balance,^13^^,^^14^ they might be helped by exercise or gardening programmes, which can be identified by social prescribers. Indeed, structured physical activity can reduce mobility disability,^25^ which is the disability when intervention is most effective.^26^^,^^27^ This may mean that social prescribing has the potential to promote reablement in the early stages of disability, and is being used in a preventative approach.

Difficulty managing money, including employment and benefits support, is a common reason for referral to social prescribing services. Whilst many referrals in this study will be related to general economic circumstances, they may also reflect executive dysfunction,^28^ or financial stress after the acquisition of disability,^17^ particularly as the disability employment gap is wider for older people.^29^

In this study, as well as people at the earlier stages of disability, a number of participants with ADL disability were referred for social prescribing. This may suggest that primary care professionals are taking individual experiences into account, and not simply identifying people with high-level needs for early intervention. Information on the time spent at each stage of disability before referral would provide useful insights into referral decision-making. It is also important to acknowledge that we cannot be certain that these referrals to social prescribing were making optimal use of the service. Authors have suggested that understanding of the role of social prescribing may be incomplete.^30^ A recent government report warned that patients referred for social prescribing initiatives are often unsuitable, because their health problems are too severe.^31^ In our analyses, it is likely that the time period for data collection (2021–23) was influential. During the COVID-19 pandemic, social prescribing link workers supported people with complex health needs, including many who were shielding.^32^ Social prescribing may also be receiving referrals for patients with complex needs because of resource constraints in UK primary care,^33^ and waiting lists for mental health services, for example. In this scenario, social prescribing may be used for the most complex cases, when other options have been exhausted. Longitudinal analyses would be useful to test this hypothesis and enhance our understanding of the characteristics of people being channelled through social prescribing. The level of need and complexity of problems amongst individuals referred to social prescribing have important implications for primary care link worker capacity and, more generally, for resource allocation to community services.

Lastly, our analysis found that participants in the poorest wealth quintile were referred for social prescribing. The introduction of social prescribing was a policy response to health inequalities,^34^ and there is an ongoing need to connect those who are living in poverty to appropriate sources of support.^35^ Social prescribing was also developed with the aim of encouraging self-management,^36^ which is made more difficult by socioeconomic insecurity.^34^ However, quantitative research has been unable to prove the effectiveness of social prescribing for improving patient outcomes in disadvantaged areas.^33^ We also report that participants with long-term conditions and loneliness were referred for social prescribing, though these are not new findings.^10^^,^^37^

### Limitations of this study

Our analysis was cross-sectional, thus longitudinal research is needed to see if patients remained engaged with social prescribing over time, and if this leads to an improvement in disability. We accounted for important confounders, including loneliness and long-term conditions, but were unable to adjust for other potentially important factors, such as local service availability, or patient/practitioner beliefs about behaviour change and the potential benefits of social prescribing. Indeed, there are known geographic inequalities in ‘green’ social prescribing initiatives^38^ and link worker employment.^39^ Despite the research to suggest that social prescribing is dependent on personal resources,^40^ the number of participants referred for social prescribing in our dataset was small, which precluded an assessment of how well they took up the service, or whether this differed by gender or location (e.g. urban versus rural). Moreover, we could not reliably determine whether the support that participants actually received was appropriate to their needs (for example, were individuals with ADL disability offered welfare advice, and those with mobility disability referred for exercise classes?). We also don’t know how soon participants were referred for social prescribing after the development of a particular limitation. Lastly, it is possible that the limited number of referrals in our dataset is due to some patients not realising that they had spoken with a social prescriber. For example, over the phone during the COVID-19 pandemic, patients might have thought that they were speaking to a counsellor, particularly if they had been referred for social prescribing through colloquial language, such as ‘we have someone who can support you with…’. In addition, the definition of social prescribing in ELSA does not include self-referral or linking to other NHS services (like pharmacists and physiotherapists). It covers the direct signposting to activities by general practitioners—of which there are examples in practice—without explicitly mentioning the referral to or by a social prescribing link worker, which is the more common scheme.^41^ This may, therefore, under-estimate the number of people accessing social prescribing.

## Conclusions

In this study, participants at either end of the disability spectrum, those with difficulty managing money, and the poorest were referred for social prescribing. Further research in a larger, longitudinal sample is needed to examine (i) whether this is meeting needs, (ii) whether patients remained engaged with social prescribing, (iii) the impact on demand for general practice services, and (iv) if benefits vary with the level or nature of the disability. In this context, link worker capacity, local resources and the type of referral are important to consider (i.e. are particular types of social prescribing more useful).

## Supplementary Material

Word_version_of_supplementary_data_fdaf143

## Data Availability

The English Longitudinal Study of Ageing dataset is available in a public, open-access repository and can be accessed through the UK Data Service at https://ukdataservice.ac.uk/. The data can be used after registration and acceptance of end-user licence.

## Notes

**Laurie E Davies,** Research Associate/Pharmacist

**David R Sinclair,** Senior Research Associate

**Angel Osei,** Undergraduate pharmacy dissertation student

**Andrew Kingston,** Senior Lecturer/Statistician

**Mark Adley,** Research Associate/Social Prescribing Link Worker

**Orla Whitehead,** Associate Researcher/General Practitioner

**Barbara Hanratty,** Professor of Primary Care and Public Health/General Practitioner
