# Supplementary material for: Are participants with disability referred for social prescribing? Findings from the English Longitudinal Study of Ageing
Source: J Public Health (Oxf). 2025 Nov 3;48(1):249–55. doi: 10.1093/pubmed/fdaf143 (PMC13017203; doi:10.1093/pubmed/fdaf143)
Supplement: Word_version_of_supplementary_data_fdaf143 [file word_version_of_supplementary_data_fdaf143.docx]

**Are people with disability referred for social prescribing? Findings from the English Longitudinal Study of Ageing**

**Supplementary data**

**Contents:**

- Appendix Table 1: Definitions of confounding variables
- Appendix Table 2: The number of participants with each disability item

## Appendix Table 1: Definitions of confounding variables

| Loneliness | Loneliness was measured using the three-item University of California, Los Angeles (UCLA) Loneliness Scale.^1^ Participants were asked, on a three-point scale of ‘never or hardly ever’, ‘some of the time’ or ‘often’, how often they felt left out, isolated from others, or that they lack companionship. A score of six or more was classed as lonely. |
| --- | --- |
| Disease count | Disease count was measured by response to ever having had any of the following self-reported doctor diagnoses: coronary heart disease (angina/myocardial infarction, high cholesterol), stroke, long covid, chronic pain,^2^ hypertension, other cardiovascular disease (heart failure, heart murmur, atrial fibrillation), diabetes, arthritis, osteoporosis, cancer, respiratory disease (asthma or chronic lung disease), depression (CES-D≥3),^3^ Alzheimer’s disease or dementia, or an emotional nervous or psychiatric problem. |
| Socioeconomic position | We used total-net non-pension wealth as a measure of socioeconomic position,^4^ and cut the variable ‘nettotw_bu_s’ into quintiles based on the whole sample. |

**References**

1. Hughes ME, Waite LJ, Hawkley LC, Cacioppo JT. A Short Scale for Measuring Loneliness in Large Surveys: Results From Two Population-Based Studies. Research on Aging 2004; 26(6): 655-72.
2. Davies LE, Spiers GF, Sinclair DR, Kingston A, Hanratty B. Characteristics of older unpaid carers in England: a study of social patterning from the English Longitudinal Study of Ageing. Age Ageing 2024; 53(3): afae049.
3. Radloff LS. The CES-D Scale: A Self-Report Depression Scale for Research in the General Population. Applied Psychological Measurement 1977; 1(3): 385-401.
4. Demakakos P, Biddulph JP, Bobak M, Marmot MG. Wealth and mortality at older ages: a prospective cohort study. Journal of Epidemiology and Community Health 2016; 70(4): 346.

## Appendix Table 2: The number of participants with each disability item

|  |  | Sample size, % (n) |
| --- | --- | --- |
| Binary disability items | Mobility | 35.1 (1960/5578) |
|  | IADL | 14.8 (828/5578) |
|  | ADL | 15.8 (882/5578) |
| Individual disability items | Dressing | 12.1 (673/5578) |
|  | Walking across a room | 3.4 (192/5578) |
|  | Bathing | 7.0 (398/5578) |
|  | Eating | 2.0 (113/5578) |
|  | Getting in/out bed | 5.9 (329/5578) |
|  | Going to the toilet | 4.1 (227/5578) |
|  | Preparing a meal | 4.0 (222/5578) |
|  | Shopping | 6.8 (382/5578) |
|  | Taking medication | 1.3 (75/5578) |
|  | Doing work around the house/garden | 12.9 (719/5578) |
|  | Managing money | 2.1 (116/5578) |
|  | Climbing stairs | 29.5 (1645/5578) |
|  | Walking quarter mile | 25.1 (1399/5578) |
| Sensitivity analysis for housework | Pushing/pulling large objects (heavy housework proxy i.e. vacuuming) | 14.2 (794/5578) |
|  | Reaching or extending arms above shoulder level (light housework proxy i.e. dusting) | 9.2 (513/5578) |
| Proxy for difficulty cutting toenails | Difficulty stooping/bending/crouching | 35.7 (1992/5578) |
